# The absence of oestrogen receptor beta disturbs collagen I type deposition during Achilles tendon healing by regulating the IRF5‐CCL3 axis

**DOI:** 10.1111/jcmm.15592

**Published:** 2020-08-10

**Authors:** Xuting Bian, Tianyao Liu, Mingyu Yang, Chengyi Gu, Gang He, Mei Zhou, Hong Tang, Kang Lu, Fan Lai, Feng Wang, Qiandong Yang, Jan‐Åke Gustafsson, Xiaotang Fan, Kanglai Tang

**Affiliations:** ^1^ Department of Orthopedic Surgery Southwest Hospital Army Medical University Chongqing China; ^2^ Department of Developmental Neuropsychology School of Psychology Third Military Medical University, Army Medical University Chongqing China; ^3^ Department of Orthopedic Surgery Affiliated Renhe Hospital of China Three Gorges University China Three Gorges University Yichang China; ^4^ Center for Nuclear Receptors and Cell Signaling University of Houston Houston TX USA; ^5^ Center for Innovative Medicine Department of Biosciences and Nutrition Karolinska Institute Novum Sweden

**Keywords:** Achilles tendon healing, collagen type I, IRF5‐CCL3 axis, oestrogen receptor β

## Abstract

Achilles tendon healing (ATH) remains an unanswered question in the field of sports medicine because it does not produce tissue with homology to the previously uninjured tissue. Oestrogen receptor β (ERβ) is involved in the injury and repair processes of tendons. Our previous study confirmed that ERβ plays a role in the early stage of ATH by affecting adipogenesis, but its role in extracellular matrix (ECM) remodelling is unknown. We established a 4‐week Achilles tendon repair model to investigate the mechanism through which ERβ affects ATH at the very beginning of ECM remodelling phase. In vitro studies were performed using tendon‐derived stem cells (TDSCs) due to their promising role in tendon healing. Behavioural and biomechanical tests revealed that ERβ‐deficient mice exhibit weaker mobility and inferior biomechanical properties, and immunofluorescence staining and qRT‐PCR showed that these mice exhibited an erroneous ECM composition, as mainly characterized by decreased collagen type I (Col I) deposition. The changes in gene expression profiles between ERβ‐knockout and WT mice at 1 week were analysed by RNA sequencing to identify factors affecting Col I deposition. The results highlighted the IRF5‐CCL3 axis, and this finding was verified with CCL3‐treated TDSCs. These findings revealed that ERβ regulates Col I deposition during ATH via the IRF5‐CCL3 axis.

## 1 INTRODUCTION

Complete rupture of the Achilles tendon is an injury that has been recognized since the time of Hippocrates and is among the more common injuries seen by sports medicine physicians.^1^ It usually occurs as a result of a sudden contraction of the gastrocnemius‐soleus muscle complex, which most often happens during sports activities, including basketball, tennis and soccer.^2^ Complete rupture of the Achilles tendon often occurs in a hypo‐vascular area 2‐6 cm proximal to the insertion on the calcaneus, and doctors have two choices for treating this injury: nonoperative treatment and surgical repair. Surgical repair, which maximizes the chances of achieving a normal or near‐normal state in the injured tendon, is considered for active and athletic patients who are more likely to perform more athletic activities after the repaid process. However, the surgical treatment of an injured tendon fails to reach the expected goal in most cases.^3^

The dry mass of human tendons is approximately 30% of the total tendon mass, and water accounts for the other 70%. In addition, 65%‐80% of the dry mass of tendons consists of collagen type I (Col I). Tenocytes, fibroblasts and tendon‐derived stem cells (TDSCs) lie between the collagen fibres along the axis of the tendon.^4^ As the main component of the extracellular matrix (ECM) of tendons, Col I imparts substantial mechanical strength to connective tissues, and thus, its recovery is of great importance to the repair of ruptured tendons.

Oestrogen receptor β (ERβ), as one of the nuclear receptors of oestrogen, was first found to be prevalent in tendon tissue in 2010.^5^ Our previous study revealed that the absence of ERβ leads to an abnormal healing process that is mainly characterized by increased adipocyte accumulation during the early repair stage of the mouse Achilles tendon, which involves augmented PPARγ signalling.^6^ Most studies on the relationship between oestrogen and Col I have focused on the effect on the collagen content in skin. Most of the results from these studies showed that oestrogen exerts a positive effect on the collagen content,^7^, ^8^, ^9^ and the addition of oestrogen to tendon tissue has a positive effect on overall collagen synthesis.^10^, ^11^

The natural tendon healing process after complete rupture typically involves a sequential series of inflammation, proliferation and remodelling.^12^ The initial step induces the release of growth factors and potent proinflammatory cytokines that recruit inflammatory cells to the site of injury. The recruited macrophages are responsible for the phagocytosis of surrounding fragments and play a key role in the proliferation of tenocytes, including TDSCs, to regulate the ECM composition, particularly the deposition of Col I.^13^, ^14^ IRF5 is a type of nuclear transcription factor and serves as a specific marker of inflammatory macrophages.^15^, ^16^ CCL3 is an important molecule downstream of IRF5. Previous studies have revealed a positive interaction between CCL3 and collagen synthesis.^17^, ^18^

In this study, we found that after 4 weeks of repair following complete rupture, the Achilles tendons of ERβ^−/−^ mice exhibited significantly weakened biomechanical properties, which corresponded to worsened mobility, compared with those of the WT controls. Observations of the tendon mass revealed that the Achilles tendons of ERβ^−/−^ mice exhibited an abnormal ECM composition profile that was mainly characterized by a decreased deposition of Col I. Furthermore, mRNA sequence analysis and bioinformatic of 1‐week repairing tendons predictions showed the involvement of IRF5‐CCL3 in the process through which the absence of ERβ leads to poor repair of injured Achilles tendons.

## 2 MATERIALS AND METHODS

### 2.1 Animal model and surgical procedure

Male ERβ^−/−^ mice and their WT littermates were used in this study. The process used to generate the ERβ^−/−^ mice on the C57BL/6J background and their primary phenotype was described by Krege et al in 1998.^19^ All the mice were housed in a temperature‐controlled room at the Third Military Medical University with a standard 12‐hour light/12‐hour dark cycle and given ad libitum access to food and water. All the experimental procedures were approved by the Third Military Medical University and performed according to the established guidelines for the care and use of laboratory animals. The following surgical procedures were performed as previously described by Palmes et al^20^ using 6‐month‐old mice with a mature skeletal system: all operations were performed under 1.00%‐2.50% isoflurane inhalation anaesthesia by using R500 series anaesthesia machine from RWD. After stable anaesthesia, both Achilles tendons of mice were exposed from their origin at the gastrocnemius muscle to their insertion into the calcaneum. After resecting the tendon of the plantaris muscle, the Achilles tendons were transected from its medial position by a standardized procedure. Then, the ends of the tendons were re‐adapted with a Kirchmayr‐Kessler suture (6‐0 Dermalon). Finally, after closing the skin, the movement of the ankles was restricted by a simple external fixation remodelled from 10 000 mL‐tips for the first two days after operation to avoid suture failure due to overstretching of the operated tendons. After the operation, the mice were given 1 or 4 weeks to allow tendon repair and were then sacrificed. The whole hind limb, including the gastrocnemius‐Achilles tendon‐calcaneus complex, was retained for further processing in case of unnecessary damage to tendons. After being fixed, the sections of repaired tissues were used for further analysis.

### 2.2 Cell isolation, culture and reagents

Tendon‐derived stem cells were isolated from 6‐week‐old male Sprague‐Dawley rats as previously described.^21^ The Achilles tendon tissues were dissected to collect only the mid‐substance tissue, and the peritendinous connective tissue was carefully removed. The collective tissues were then washed with 0.01 mol/L PBS and digested in 3 mg/mL type I collagenase (Sigma‐Aldrich) for 2 hours at 37°C. The cells were then filtered through 7‐μm nylon mesh (Becton Dickinson) to yield a single‐cell suspension. The cells were then washed in 0.01 mol/L PBS, centrifuged at 1000 rpm for 5 minutes and resuspended in fresh culture media consisting of Dulbecco's modified Eagle's medium (DMEM; Gibco) with 10% foetal bovine serum (FBS) and 1% penicillin/streptomycin (Pen/Strep) (all from Invitrogen). The TDSCs were grown at 37°C in the presence of 5% CO_2_ and passaged once they reached 70% confluence; the culture media were changed every three days. Cells from passages 2‐3 were used in the experiments. The TDCSs were seeded in 24‐well plates for cell staining and in six‐well plates for protein and RNA extraction. The identification of TDSCs is shown in Figure S3 according to Bi et al.^22^ The TDSCs were stimulated with CCL3 (HY‐P7255, MCE) at a concentration of 200 µg/L in 0.01 mol/L PBS according to Zhang et al.^23^

### 2.3 Histomorphometry and cellular morphometry

The tendons were fixed in 4% buffered formalin at 4°C for 24 hours and then in 30% sucrose at 4°C for 24 hours, dehydrated, embedded in optimal cutting temperature compound (OCT) and processed to obtain longitudinal sections (7 μm). The histology of the Achilles tendon in the defect zone was assessed by haematoxylin‐eosin (HE) staining and then graded by two blinded investigators using the histological score system given in Table S1, which was based on that proposed by Stoll et al^24^ and modified by Lin et al.^25^ The collagen content in tendon scars was evaluated by Sirius Red staining. Immunohistochemistry and immunofluorescence were performed according to Bian et al.^6^ The sections were incubated with 0.3% Triton X‐100 in phosphate‐buffered saline for 30 minutes at 37°C. The sections were then incubated with 3% bovine serum albumin (BSA) for 30 minutes at 37°C to block nonspecific binding and then with primary antibodies against collagen I (ab34710; Abcam), collagen III (ab; Abcam), IRF5 (ab181553; Abcam), CCL3 (ab25128; Abcam), and ERβ (PA1‐313; Invitrogen) diluted in 1% BSA and 0.1% Triton X‐100 for 2 hours at 37°C and then overnight at 4°C; sections incubated with 1% BSA and 0.1% Triton X‐100 were used as negative controls. The next day, the sections were washed with 0.01 mol/L phosphate‐buffered saline (PBS) and incubated with biotin‐conjugated secondary antibodies, Cy3‐conjugated donkey anti‐rabbit secondary antibodies or Alexa Fluor 488‐conjugated goat anti‐mouse antibodies for 2 hours at 37°C.

All the stained sections and cells were viewed and photographed under a Zeiss AxioVert microscope equipped with a Zeiss AxioCam digital colour camera connected to the Zeiss AxioVision 3.0 system.

### 2.4 Quantitative reverse transcriptase polymerase chain reaction

Total RNA from tendon tissues was isolated using a kit (Beyotime Institute of Biotechnology) and utilized for qRT‐PCR. Briefly, the PCRs were pipetted on ice, and each well contained 2.5 μL of cDNA, 2.5 μL of the primers and 5 μL of iTaq™ Universal SYBR Green SuperMix. The plates were subsequently sealed, centrifuged for 20 seconds at 1400 rpm, incubated at 95°C for 5 minutes and then subjected to 30 cycles of a three‐step temperature program consisting of 1 minute at 95°C, 20 seconds at 65°C and 30 seconds at 72°C. The relative gene expression levels were quantified by densitometry, normalized to the level of the housekeeping gene glyceraldehyde 3‐phosphate dehydrogenase (GAPDH) and presented as fold‐changes relative to the WT controls. All PCR results were reproduced independently in five experiments. The primer sequences used in these experiments are listed in Table S2.

### 2.5 Western blot analysis

The Achilles tendons from both groups and TDSCs were isolated and homogenized in ice‐cold RIPA lysis buffer (Beyotime). After centrifugation of the lysates at 12 000 rpm and 4°C for 15 minutes, the protein concentration was determined using a bicinchoninic acid kit (Beyotime Institute of Biotechnology). The protein samples (20 μg/lane) were separated on a 12% SDS‐polyacrylamide gel for 50 minutes at 120 V and then transferred to a nitrocellulose membrane for 70 minutes at 120 V. The membranes were blocked with Tris‐buffered saline containing 0.1% Tween 20 and 5% fat‐free milk for 2 hours at RT. The membranes were then incubated overnight at 4°C with rabbit antibodies against Col I (ab34710; Abcam) and IRF5 (ab181553; Abcam) that can react with mouse and rat and then incubated for 2 hours at RT with peroxidase‐conjugated goat anti‐rabbit immunoglobulin G antibody (1:1000; Santa Cruz Biotechnology). For all the antibodies, the NC membranes were then scanned using the Odyssey infrared imaging system with Odyssey Application software V1.2.15. All Western blotting data are representative of at least five independent experiments.

### 2.6 RNA‐seq and data analysis

Total RNA was extracted from the Achilles tendons after 1‐week repairing from both ERβ^−/−^ and WT mice using Trizol (Invitrogen). Total RNA was qualified and quantified using a Nano Drop and Agilent 2100 bioanalyzer (Thermo Fisher Scientific). Oligo(dT)‐attached magnetic beads were used to purified mRNA. Then, First‐strand cDNA was generated in First‐Strand reaction system by PCR, and the second‐strand cDNA was generated as well. The cDNA fragments with adapters were amplified by PCR, and the products were purified by Ampure XP Beads. The library was assessed quality and quantity using the Agilent 2100 bioanalyzer, then, undergoes DSN treatment. The DSN treated library was assessed quality to ensure the high quality of the sequencing data. The qualified library was amplified on cBot to generate the cluster on the flowcell. And the amplified flowcell was sequenced single end on the HiSeq4000 (BGI). Reference genome using Hisat2 software was used to align the trimmed reads. StringTie was used to estimate the transcript abundances of each sample. R package Ballgown was used to calculate the FPKM value for differentially expressed genes and transcripts. StringTie and Ballgown were used to predict novel genes and transcripts, and CPAT was used to assess the coding potential of those sequences. rMATS.42 Principle Component Analysis was used to detect Alternative splicing events and plots, and gene expression level was used for correlation analysis.

### 2.7 Behavioural and biomechanical tests

An open‐field behavioural test was performed and evaluated as previously described by Xiao et al.^26^ The open‐field apparatus was constructed of grey plywood and measured 40 × 40 cm with 30‐cm‐high walls. The mice were placed in the centre of the open field, and the movements of each mouse were recorded for 10 minutes for further analysis by using a video camera secured to the top of the apparatus and analysed using EthoVision 11.0 (Noldus). Before measurements were taken, every mouse was put into the apparatus for 10‐minute acclimation. After each subject, the test apparatus was cleaned with 70% ethanol and wiped out with clean paper towels. The distance travelled (cm) and speed (cm/s) were recorded to reflect the basic motor ability of the mice.

Biomechanical tests were performed according to Muller et al.^27^ The tendon length (mm) and cross‐sectional area (mm^2^) were measured using a high‐precision calibre which has the accuracy of 0.02 mm, and biomechanical tests were then performed using a mechanical testing machine (ElectroForce, 5500 Series) containing clamps to which the calcaneus and gastrocnemius muscle belly were fastened. The tendons were maintained moist with Ringer's solution throughout the test process. The displacement rate was constantly set to 1000 mm/min. The force displacement rates were registered digitally and subsequently analysed automatically by the machine. The maximal Stress to failure (N) and tendon stiffness (N/mm) were measured. The tendon stiffness (N/mm) was calculated from the linear part of the force‐elongation curve. The elastic modulus (MPa) was calculated using the following equation: *E* = *FL*/*S*Δ*L*, where *F* is the maximal stress to failure, *L* is the length of the Achilles tendon, *S* is the cross‐sectional area of the Achilles tendon, and Δ*L* is the change in length with the maximal stress to failure.

### 2.8 Statistical analysis

The statistical analyses were performed using SPSS 25.0 software (SPSS Inc). The statistical significance of the differences between two groups was determined using the two‐tailed unpaired Student's *t* test, the significance of the differences between two groups among different timing was analysed by two‐way ANOVA, and the significance of the differences among three or five groups was assessed with the two‐tailed nonparametric Mann‐Whitney test or one‐way analysis of variance followed by Fisher's protected least significant difference post hoc test. The sample size and experimental replications are indicated for each method. The results are presented as the means ± SDs. Differences reached statistical significance at *P < .05, **P < .01 and ***P < .001.

## 3 RESULTS

### 3.1 After 4 weeks of repair, ERβ^−/−^ mice exhibit weaker sport ability compared with WT controls

**Figure 1:**
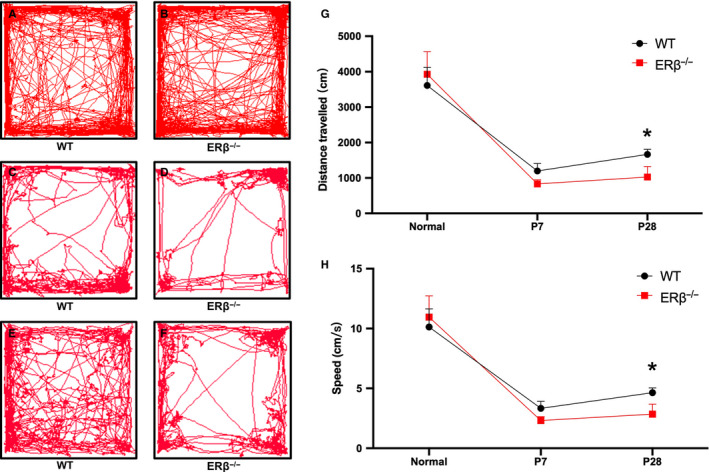
ERβ^−/−^ mice exhibited poorer performance in the open‐field test after 4 wk of repair compared with WT control mice. A and B, Illustrative example of the travel pathways of a pair of normal WT and ERβ^−/−^ mice in the open‐field test. C and D, The distance travelled and speed of the mice in the open‐field test showed no difference between the two groups. E and F, Illustrative example of the travel pathways of a pair of WT and ERβ^−/−^ mice after 1 wk of repair in the open‐field test. G, Statistic analysis of distance travelled between WT and ERβ^−/−^ among native status, 1 wk after injury and 4 wk after injury by two‐way ANOVA. After 4‐wk repairing, the distance travelled of ERβ^−/−^ was significantly shorter than that of WT. H, Statistic analysis of speed between WT and ERβ^−/−^ among native status, 1 wk after injury and 4 wk after injury by two‐way ANOVA. After 4‐wk repairing, the speed of ERβ^−/−^ was significantly slow than that of WT. The data are presented as the means ± SDs (n = 5‐8); *P < .05

The mobility of ERβ^−/−^ and WT control mice was assessed through a 10‐minute open‐field test, and the results showed no significant differences in the distance travelled (cm) and speed (cm/s) between the two groups (Figure 1A, B, G, H). One week after injury, which represents the early healing stage, the sport ability of both the ERβ^−/−^ and WT control mice was significantly weakened, as reflected by marked decreases in the distance travelled and speed. In addition, the ERβ^−/−^ mice exhibited notably worse performance (Figure 1C, D, G, H). Four weeks after injury, the distance travelled and speed of the WT mice showed partial recovery compared with those measured 1 week after injury but remained worse than those of normal mice (Figure 1A, C, E, G, H). However, both the distance travelled and the speed of the ERβ^−/−^ mice exhibited nearly no improvement 4 weeks after injury compared with 1 week after injury (Figure 1D, F, G, H). These results showed that the sport ability of both ERβ^−/−^ and WT control mice was obviously weakened after Achilles tendon injury, but the ERβ^−/−^ mice exhibited a delayed repair process after 4 weeks.

### 3.2 The tendon scars of ERβ^−/−^ mice exhibit inferior biomechanical properties after 4 weeks of repair compared with those of WT control mice

**Figure 2:**
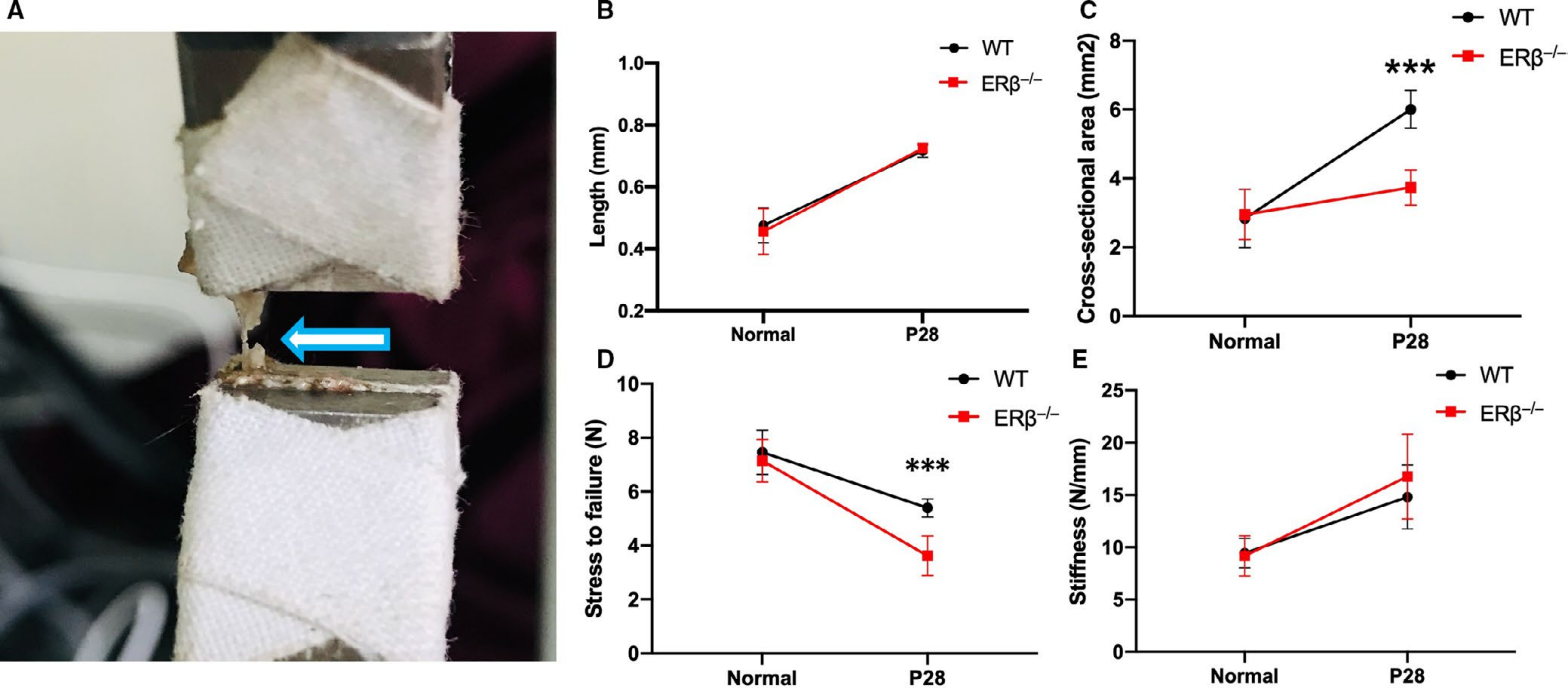
ERβ^−/−^ tendon scars exhibit inferior biomechanical properties after 4 wk of healing compared with WT control tendon scars. A, Illustrative example of a biomechanical test of an Achilles tendon after 4 wk of repair. B, No difference in length was found between the WT and ERβ^−/−^ Achilles tendons, but these were longer than the NTs. C, ERβ^−/−^ Achilles tendons has significantly smaller cross‐sectional areas than WT control tendons, and both of these tendons have larger cross‐sectional areas than NTs. D, ERβ^−/−^ Achilles tendons are weaker than WT control tendons, and both ERβ^−/−^ and WT control Achilles tendons are weaker than NTs. E, No difference in stiffness was found between the WT and ERβ^−/−^ Achilles tendons, but these tendons are stiffer than NTs. The data are presented as the means ± SDs (n = 5); ***P < .001

First, for the analysis of biomechanical properties, we analysed the length (mm), cross‐sectional area (mm^2^), maximal stress to failure (N) and tendon stiffness (N/mm) of the normal Achilles tendons of ERβ^−/−^ and WT control mice. The results found no significant difference in any of these four variables between the two groups (Figures S1‐S4). The normal WT group was used to obtain the native contralateral tendons (NTs), which are shown as a horizontal line in the figures. We subsequently continued to observe whether the variables showed changes after 4 weeks of repair. Both the WT and ERβ^−/−^ tendons were significantly longer after 4 weeks of repair compared with the NTs, but no difference was found between the two groups (Figure 2B). The analysis of the cross‐sectional areas showed that both the WT and ERβ^−/−^ tendons were significantly larger after 4 weeks of repair than the NTs, and after 4 weeks of repair, the ERβ^−/−^ tendons exhibited markedly smaller cross‐sectional areas than the WT tendons (Figure 2C). The tear resistance of the ERβ^−/−^ tendons was significantly lower than that of the WT controls. The mean stress to failure of the NTs was 7.46 ± 0.82 N, and the tendons from both groups of mice were significantly weaker than the NTs after 4 weeks of repair (Figure 2D). The tendons appeared to exhibit significantly greater stiffness than the NTs after 4 weeks of repair, and no difference was found between the two groups (Figure 2E). These results indicated that the biomechanical properties of the ERβ^−/−^ tendons were inferior compared with those of the WT control tendons after a 4‐week repair process.

### 3.3 After repair, the ERβ^−/−^ tendons exhibit erroneous collagen deposition

**Figure 3:**
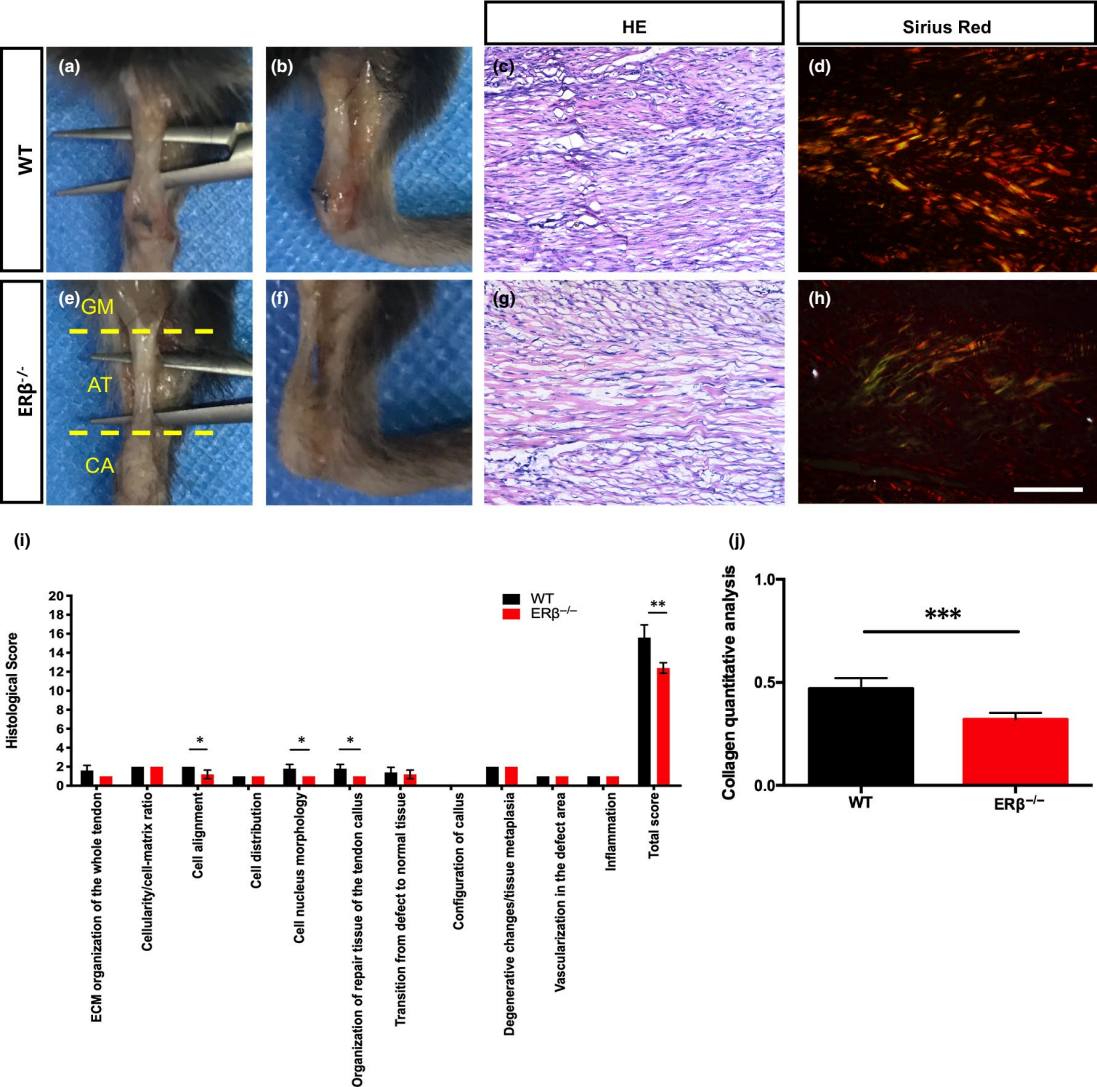
ERβ^−/−^ tendons exhibit poor repair, and this process is characterized by erroneous collagen deposition. A‐D, Appearance of WT and ERβ^−/−^ Achilles tendons after 4 wk of repair. E‐G, HE staining‐based evaluation of tendon healing using an established histological scoring system revealed significantly lower histological scores for ERβ^−/−^ mice compared with WT control mice after 4 wk of repair. H‐J, Sirius Red staining and collagen quantitative analysis revealed decreased collagen deposition in ERβ^−/−^ Achilles tendons than WT control tendons after 4 wk of repair. The data are presented as the means ± SDs (n = 5); **P < .01, ***P < .001. Scale bars: C, D, G, H: 100 μm

After identifying the above‐described changes in mobility and biomechanical properties, we assessed the histomorphology of the tendons during the repair process. A visible distinction in appearance was found between the two groups after 4 weeks of repair: the ERβ^−/−^ tendons were thinner than the WT tendons (Figure 3A‐D). Haematoxylin and eosin staining revealed that the sectioned ERβ^−/−^ tendons exhibited a significantly different tissue organization, as manifested by significantly inferior total histological scores, compared with the WT control tendons (Figure 3E‐G). Furthermore, the interstitial collagen content of the tendons was evaluated by Sirius red staining (Figure 3H, I), and the statistical analysis of scanned images using Zeiss AxioVision 3.0 software revealed that the collagen fibre in the ERβ^−/−^ tendons was significantly lower than that of the WT control tendons (Figure 3J). These histomorphological results revealed that the functional defect in ERβ^−/−^ tendons was related to a reduced collagen content.

### 3.4 ERβ^−/−^ tendon scars show an abnormal ECM composition manifested by an aberrant collagen I content

**Figure 4:**
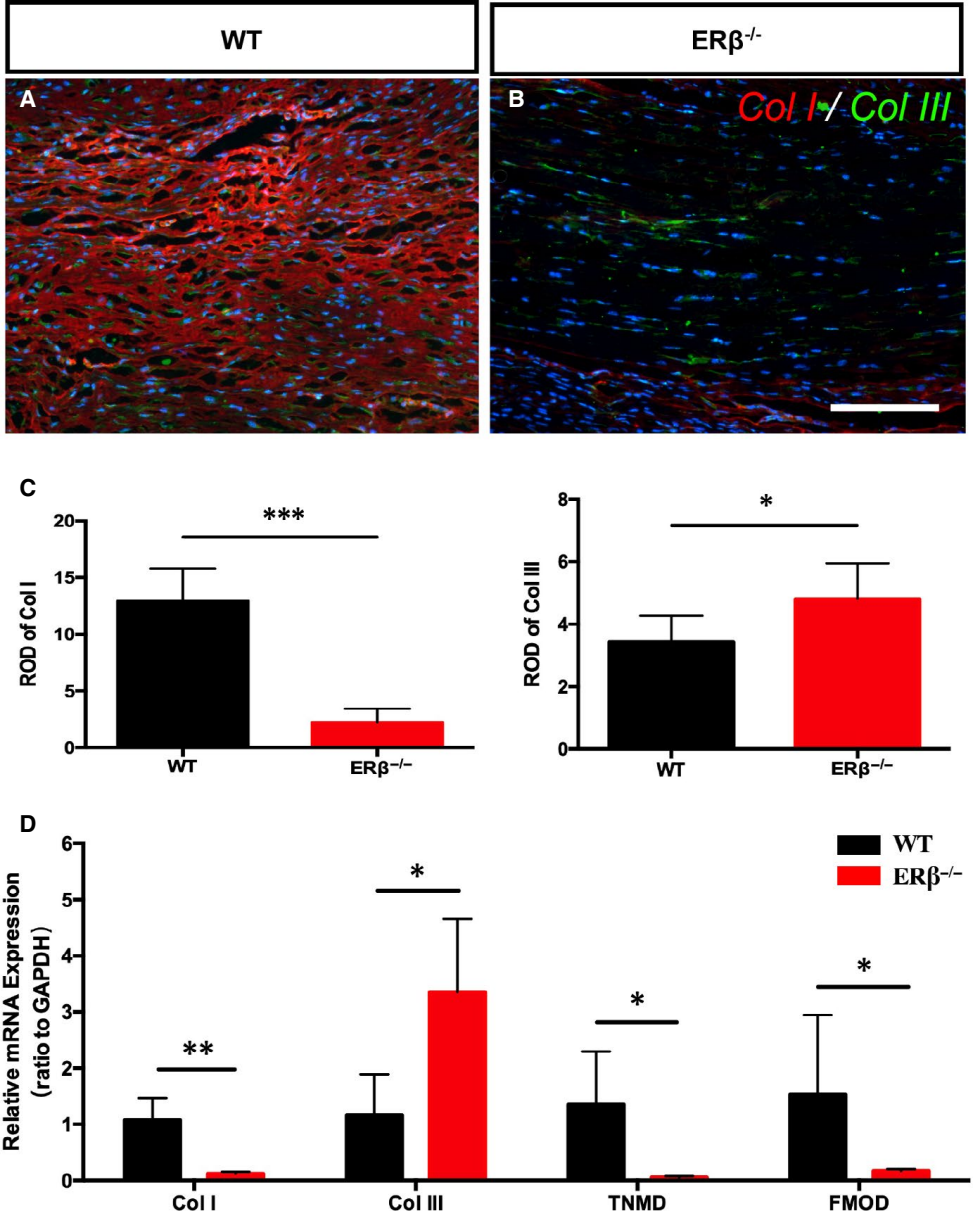
ERβ^−/−^ tendon scars show abnormal ECM composition mainly characterized by abnormal collagen I levels. A and B, Immunofluorescence analysis of collagen I (red) and III (green). C and D, Statistical analysis of relative optical density (ROD) revealed that the ERβ^−/−^ tendons contain significantly less Col I and slightly more Col III after 4 wk of repair. E, qRT‐PCR data showed significantly lower Col I mRNA expression, higher Col III mRNA expression and slightly decreased TNMD and FMOD mRNA expression. The data are presented as the means ± SDs (n = 5); *P < .05, **P < .01, and ***P < .001. Scale bars: 100 μm

To further investigate the mechanism through which the absence of ERβ affects collagen deposition, we performed an immunofluorescence analysis of Col I and Col III, which are the two main types of collagen found in both normal and injured tendons (Figure 4A, B). The statistical analysis showed that the ERβ^−/−^ tendons contained significantly less Col I and slightly more Col III 4 weeks of repair (Figure 4C, D). We also examined the gene expression of Col I and Col III as well as two other components of the ECM (TNMD and FMOD) at the mRNA level, and the results showed that the changes in the protein deposition of Col I and Col III in the ERβ^−/−^ tendons were consistent with the qRT‐PCR data (lower Col I mRNA levels and higher Col III mRNA levels in the ERβ^−/−^ tendons). Slight decreases in the mRNA expression levels of TNMD and FMOD were also detected in the ERβ^−/−^ tendons (Figure 4E). These results revealed that ERβ^−/−^ tendon scars exhibit an abnormal ECM composition, as mainly characterized by decreased Col I deposition, after 4 weeks of repair and confirmed our initial chemical staining results.

### 3.5 RNA‐seq analysis of the gene expression profile of ERβ^−/−^ and WT control tendon scars revealed IRF5 down‐regulation in the absence of ERβ

**Figure 5:**
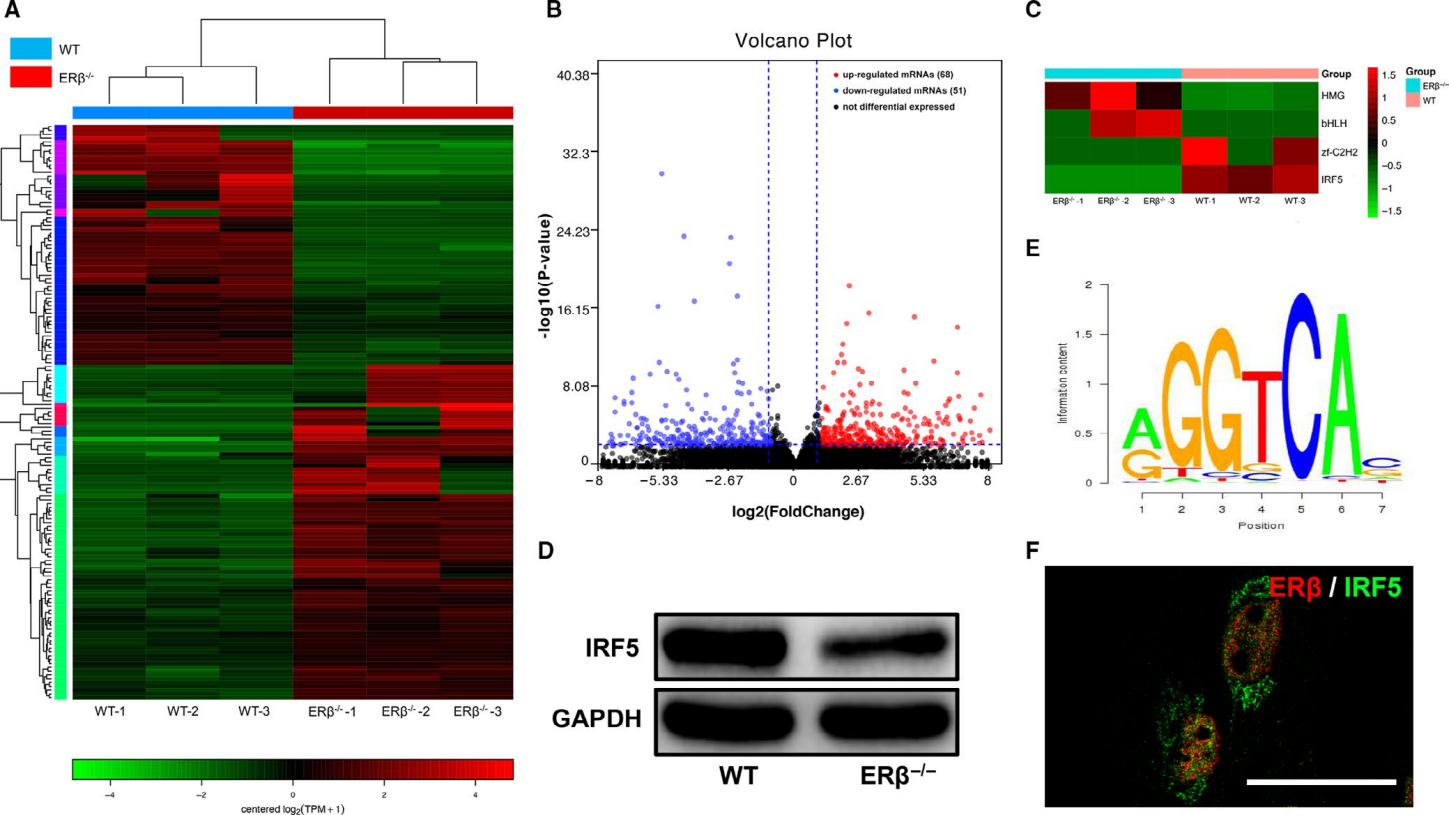
RNA‐seq analysis of the gene expression profile of ERβ^−/−^ and WT control tendon scars revealed IRF5 down‐regulation in the absence of ERβ. A, Heatmap depicting the expression levels of genes in WT and ERβ^−/−^ tendons after 1 wk of repair. B, Volcano map of differentially expressed genes between WT and ERβ^−/−^ tendons after 1 wk of repair: 68 genes were up‐regulated, and 51 genes were down‐regulated. C, Heatmap of differentially expressed transcription factors. D, Immunoblots of IRF5 in WT and ERβ^−/−^ tendons after 1 wk of repair. E, Bioinformatic prediction of binding sites of ERβ and IRF5 using JASPAR (chr6:29528943‐29528949, AGGGCAG, length = 7). F, The immunofluorescence analysis of ERβ and IRF5 revealed co‐expression in macrophages. Scale bars: 50 μm

The changes in the gene expression profiles of tendons after 1 week of repair were detected by RNA sequencing to analyse the mechanism underlying the changes observed in tendons after 4 weeks of repair related to the above‐described abnormal deposition of collagen. The early healing stage of the injured tendons consists of an inflammatory response that is closely related to ECM production and remodelling. The heatmap and volcano map of the RNA sequencing results (Figure 5A, B) revealed that 68 genes and 51 genes with log^2^ ratios higher than 2 were up‐ and down‐regulated, respectively, in the ERβ^−/−^ tendons compared with the WT control tendons. To identify a mechanism of action involving transcriptional regulation, we subsequently focused on differentially expressed transcription factors because ERβ is also a type of transcription factor. As shown in the heatmap, the analysis of differentially expressed transcription factors identified two up‐regulated (HMG and bHLH) and down‐regulated (zf‐C2H2 and IRF5) transcription factors (Figure 5C). The prediction of the transcription factor binding profiles using SwissRegulon Portal revealed that ERβ likely binds to IRF5, and the predicted binding site information was the following: chr6:29528943‐29528949, AGGGCAG, length = 7 (Figure 5E). We subsequently confirmed that the protein expression of IRF5 was decreased in the ERβ^−/−^ tendons by WB (Figure 5D). A subsequent immunofluorescence analysis of ERβ and IRF5 revealed their co‐expression in TDSCs (Figure 5F). All these results revealed that IRF5 might be involved in the process through which the absence of ERβ affects the repair of tendons.

### 3.6 The IRF5‐CCL3 axis is involved in the ERβ‐regulated production of Col

**Figure 6:**
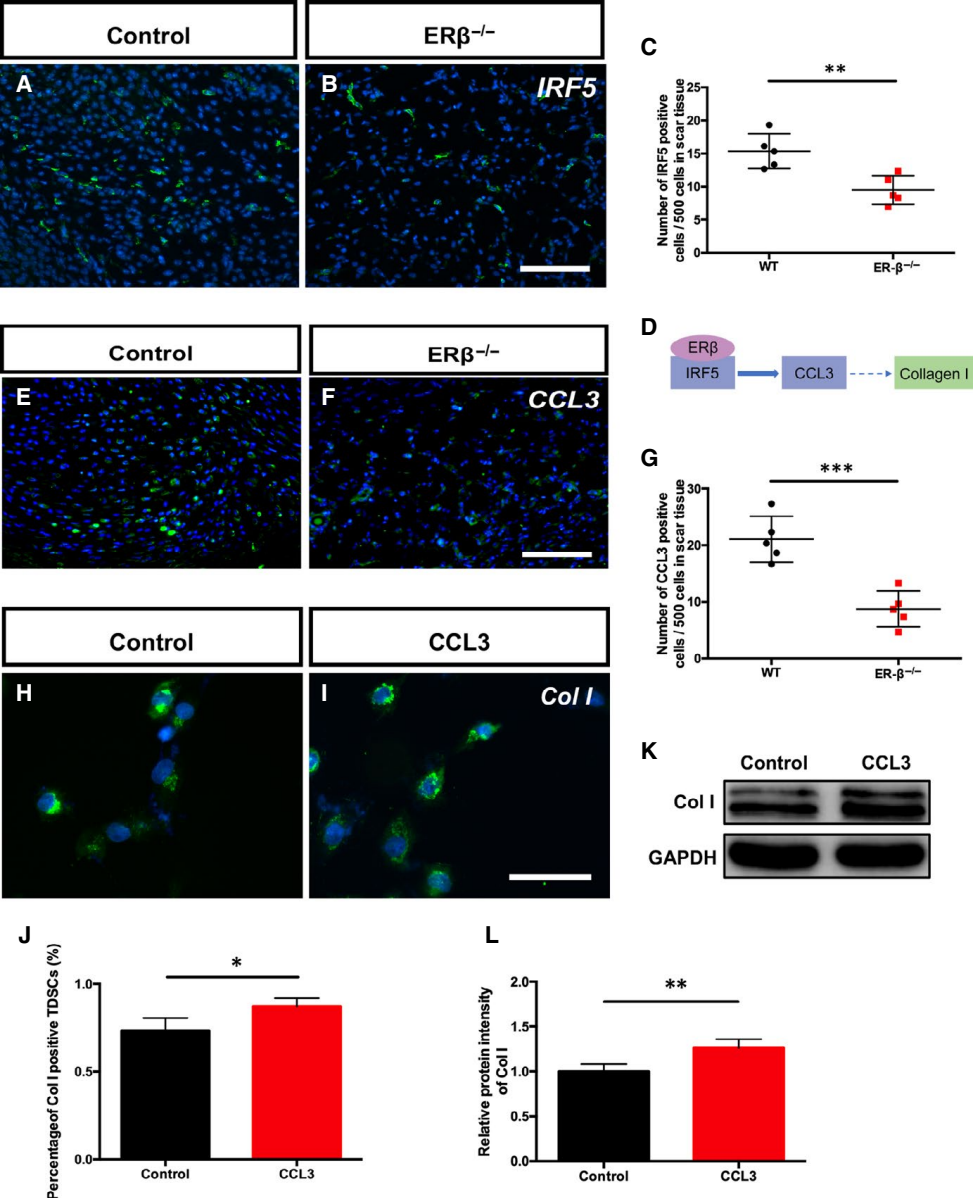
The IRF5‐CCL3 axis is involved in ERβ‐regulated production of Col. A and B, Immunofluorescence of IRF5 in tendons after 1 wk of repair. C, A statistical analysis revealed that the number of IRF5‐positive cells was significantly decreased in ERβ^−/−^ tendons compared with WT control tendons. D, Schematic diagram of the involvement of IRF5‐CCL3 in the process through which ERβ affects Col I. E and F, Immunofluorescence of CCL3 in tendons after 1 wk of repair. G, A statistical analysis revealed that the number of CCL3‐positive cells in ERβ^−/−^ tendons was significantly lower than that in the WT control tendons. H and I, Immunofluorescence of Col I in control and CCL3‐stimulated TDSCs. J, A statistical analysis of the percentage of Col I‐positive cells showed a significant increase after CCL3 stimulation. K and L, Representative immunoblots and statistical analysis of Col I in the control and CCL3‐stimulated groups. The data are presented as the means ± SDs (n = 5); *P < .05, **P < .01, and ***P < .001. Scale bars: A, B, F, G:100 μm; I, J: 50 μm

Based on the RNA‐sequence analysis and bioinformatics prediction results, we performed an immunofluorescence analysis of IRF5 in the tendons from both groups after 1 week of repair, and the statistical analysis showed that the number of IRF5‐positive cells in the ERβ^−/−^ tendons was significantly decreased compared with that in the WT control tendons (Figure 6A‐C). Previous studies have revealed that CCL3 can induce the expression of Col I in fibroblasts,^18^ and CCL3 is a key molecule downstream of IRF5 (Figure 6D). Thus, we performed an immunofluorescence analysis of CCL3 in the tendons after 1 week of repair to examine the expression of this molecule (Figure 6E, F). The statistical analysis showed that the numbers of CCL‐positive cells in the ERβ^−/−^ tendons was lower than that in the WT control tendons (Figure 6G). We subsequently examined the expression of Col I in TDSCs cultured with CCL3 for 24 hours by immunofluorescence and WB, and the results showed that the expression of Col was significantly increased after simulation with CCL3 (Figure 6H‐L). All of these results revealed that the IRF5‐CCL3 axis is involved in the ERβ‐regulated production of Col both in vivo and in vitro.

## 4 DISCUSSION

Tendons transmit force from muscle to bone and act as a buffer by absorbing external forces to limit muscle damage.^4^ Healthy tendons exhibit high mechanical strength, good flexibility and an optimal level of elasticity to perform their unique role. The mechanical behaviour of tendons depends on the levels of the various types of collagen present in the tendons. Tendon rupture is a type of tendon injury that is largely caused by an acceleration‐deceleration mechanism. The Achilles tendon, which is the largest and strongest tendon in the human body, is involved in as many as half of all sports‐related injuries. Participating in a sports activity is the most common aetiological factor resulting in injury.^28^, ^29^ Despite the remodelling process, the biomechanical properties of the healed tendon tissues never match those of the intact tendon tissues.^4^ In fact, a study of transected sheep Achilles tendons after 12 months of spontaneous healing revealed that their rupture force was only 56.7% of the normal level.^30^

Many factors affect the repair process after tendon rupture, but this problem has not been perfectly resolved. The risk of traumatic tendon injuries due to overuse differs between women and men, and some of this gender difference in injury risk is likely explained by oestrogen differences.^31^, ^32^ The effects of oestrogen have not been fully elucidated.^33^ In women, the presence of oestrogen at high levels might be beneficial during regular tissue loading or during recovery after injury because oestrogen can enhance the collagen synthesis rate in tendons.^34^, ^35^ Oestrogen primarily binds to the oestrogen receptors (α and β) in the nucleus and G‐protein‐coupled oestrogen receptor (GPER/GPR30) in the membrane.^36^, ^37^ It should be noted that oestrogen might exert different effects on the biomechanical properties of tendons due to disparities in the loading profile and differences in the relative distribution and numbers of oestrogen receptors.^38^, ^39^

Our recent study demonstrated that the absence of ERβ leads to abnormalities in Achilles tendon healing at the early stage and that these are mainly characterized by increased adipocyte accumulation.^6^ However, whether and how ERβ affects Achilles tendon repair after the early healing stage. First, we established a full‐thickness‐defect model of the mouse Achilles tendon, and the mice were given 4 weeks to allow tendon repair, which corresponds to the late phase of the repair process. Restoration of the mechanical strength is the main goal of tendon healing. Subsequently, behaviour and biomechanical tests were performed, and the results showed that the ERβ^−/−^ mice exhibited a significant reduction in open‐field activity, as reflected in both the running distance and speed, and inferior biomechanical properties, as manifested by a reduced maximal stress to failure, compared with WT control mice. Among the many substances comprising the matrix of the tendons, collagen fibres are largely responsible for the mechanical strength of this tissue.^40^ Therefore, we examined the histological appearance of both groups of tendons by HE staining and the collagen content in the tendons by Sirius Red staining after 4 weeks of repair, and the chemical staining revealed that the ERβ^−/−^ mice exhibited poorer histological scores and, more importantly, a notably reduced collagen content compared with the WT control mice. Furthermore, we performed an immunofluorescence analysis of Col I and Col III to examine whether the composition of collagen was affected. Col I imparts greater mechanical strength to connective tissues, and Col III contributes to tensile stress by decreasing the elasticity and increasing the weakness. Therefore, a decrease in the Col I‐to‐Col III ratio would have the overall effect of weakening collagen tissues.^41^ Our results showed that the ERβ^−/−^ tendons contained significantly less Col I and slightly more Col III after 4 weeks of repair. In addition, our results also revealed decreases in the TNMD and FMOD contents which were important small molecules surrounding the collagen molecules and tenocytes and participated in the repair and regeneration process in tendons in ERβ^−/−^ tendon scars after 4 weeks of repair. Together, these results suggest that the absence of ERβ leads to imperfect repair of Achilles tendons due to abnormal Col I deposition.

The modulation of events occurring during early tendon healing is essential for ECM remodelling. Therefore, we performed RNA sequencing of tendon samples after 1 week of repair, which corresponds to the early healing stage, to clarify the underlying mechanism. ERβ is a nuclear receptor that plays a role in regulating gene expression, and its binding to another nuclear transcription factor is an important factor. Therefore, we identified differentially expressed transcription factors from the set of all differentially expressed genes between the two groups. Using SwissRegulon Portal, we found that ERβ could bind to IRF5, which is the upstream molecule regulating the CCL3‐CCR5 axis. Sasaki et al^18^ found that the CCL3‐CCR5 axis is related to the numbers of Col I‐positive cells and observed decreased collagen deposition in CCL3‐ and CCR5‐deficient mice, a finding that was related to the reduced proliferation of fibroblasts. In our previous study, we also found that ERβ^−/−^ tendon scars exhibited decreased stem cell proliferation and increased cell apoptosis, and we confirmed that the activation of ERβ promoted the proliferation of TDSCs in vitro.^6^ We also cultured TDSCs with CCL3 to assess its effect on Col I production, and the results showed an increase in Col I expression. These data demonstrated that ERβ can affect Col I deposition during Achilles tendon healing by regulating the IRF5‐CCL3 axis.

However, the study of ours has the limitation that in vitro part mismatches the knockout mice due to technical reason, but from another point of view, this arrangement confirms the conclusion through different model animals.

## 5 CONCLUSIONS

Collectively, our findings in this study provide the first demonstration that the absence of ERβ leads to abnormal deposition of Col I during repair of the mouse Achilles tendon and that this process is regulated by the IRF5‐CCL3 axis.

## CONFLICT OF INTEREST

The authors confirm that there are no conflicts of interest.

## AUTHOR CONTRIBUTIONS

**Xuting Bian**: Conceptualization‐Lead, Data curation‐Lead, Formal analysis‐Lead, Investigation‐Lead, Methodology‐Lead, Project administration‐Lead, Software‐Lead, Writing‐original draft‐Lead. **Tianyao Liu**: Data curation‐Supporting, Methodology‐Supporting, Resources‐Supporting. **Mingyu Yang**: Investigation‐Supporting. **Chengyi Gu**: Investigation‐Supporting, Methodology‐Supporting. **Gang He**: Data curation‐Supporting, Formal analysis‐Supporting, Investigation‐Supporting, Methodology‐Supporting. **Mei Zhou**: Data curation‐Supporting, Investigation‐Supporting. **Hong Tang**: Data curation‐Supporting, Investigation‐Supporting, Methodology‐Supporting. **Kang Lu**: Data curation‐Supporting, Formal analysis‐Supporting, Software‐Supporting. **Fan Lai**: Investigation‐Supporting, Methodology‐Supporting. **Feng Wang**: Investigation‐Supporting, Methodology‐Supporting, Resources‐Supporting. **Qiandong Yang**: Data curation‐Supporting, Investigation‐Supporting. **JanAke Gustafsson**: Resources‐Supporting, Writing‐review & editing‐Supporting. **Xiaotang Fan**: Funding acquisition‐Supporting, Methodology‐Supporting, Project administration‐Equal, Resources‐Supporting, Supervision‐Equal, Validation‐Equal, Writing‐review & editing‐Equal. **Kanglai Tang**: Conceptualization‐Supporting, Funding acquisition‐Lead, Project administration‐Supporting, Resources‐Supporting, Supervision‐Equal, Validation‐Equal, Writing‐review & editings‐Equal.

## Supporting information

Supplementary Material

## Data Availability

All data generated or analysed during this study are included in this published article.
