# Supplementary material for: The absence of oestrogen receptor beta disturbs collagen I type deposition during Achilles tendon healing by regulating the IRF5‐CCL3 axis
Source: J Cell Mol Med. 2020 Aug 10;24(17):9925–35. doi: 10.1111/jcmm.15592 (PMC7520326; doi:10.1111/jcmm.15592)
Supplement: Supplementary file 1 — Supplementary Material [file JCMM-24-9925-s001.docx]

Additional File for

**Absence of Estrogen Receptor Beta (ERβ）Disturbs Extracellular Matrix Remodeling by Regulating Inflammation during Early Achilles Tendon Healing**

Xuting Bian, Tianyao Liu, Mei Zhou, Gang He, Yuanyuan Ma, Mingyu Yang, Youxing Shi, Yunjiao Wang, Hong Tang, Xia Kang, Jan-Åke Gustafsson, Xiaotang Fan, and Kanglai Tang

**This file includes:**

Supplementary Materials:

Fig.S1. Identification of TDSCs

Fig.S2. Length of Native contralateral tendons of WT and ERβ^-/-^ mice

Fig.S3. Cross-sectional area of Native contralateral tendons of WT and ERβ^-/-^ mice

Fig.S4. Load to failure of Native contralateral tendons of WT and ERβ^-/-^ mice

Fig.S5. Stiffness of Native contralateral tendons of WT and ERβ^-/-^ mice

Fig.S6 Heatmap of correlation analysis of RNA sequencing between WT and ERβ-/- mice

Table.S1. Histological scoring system

Table.S2. qt-PCR Primers

**Supplementary Figure 1 ——Identification of TDSCs[1]**

**
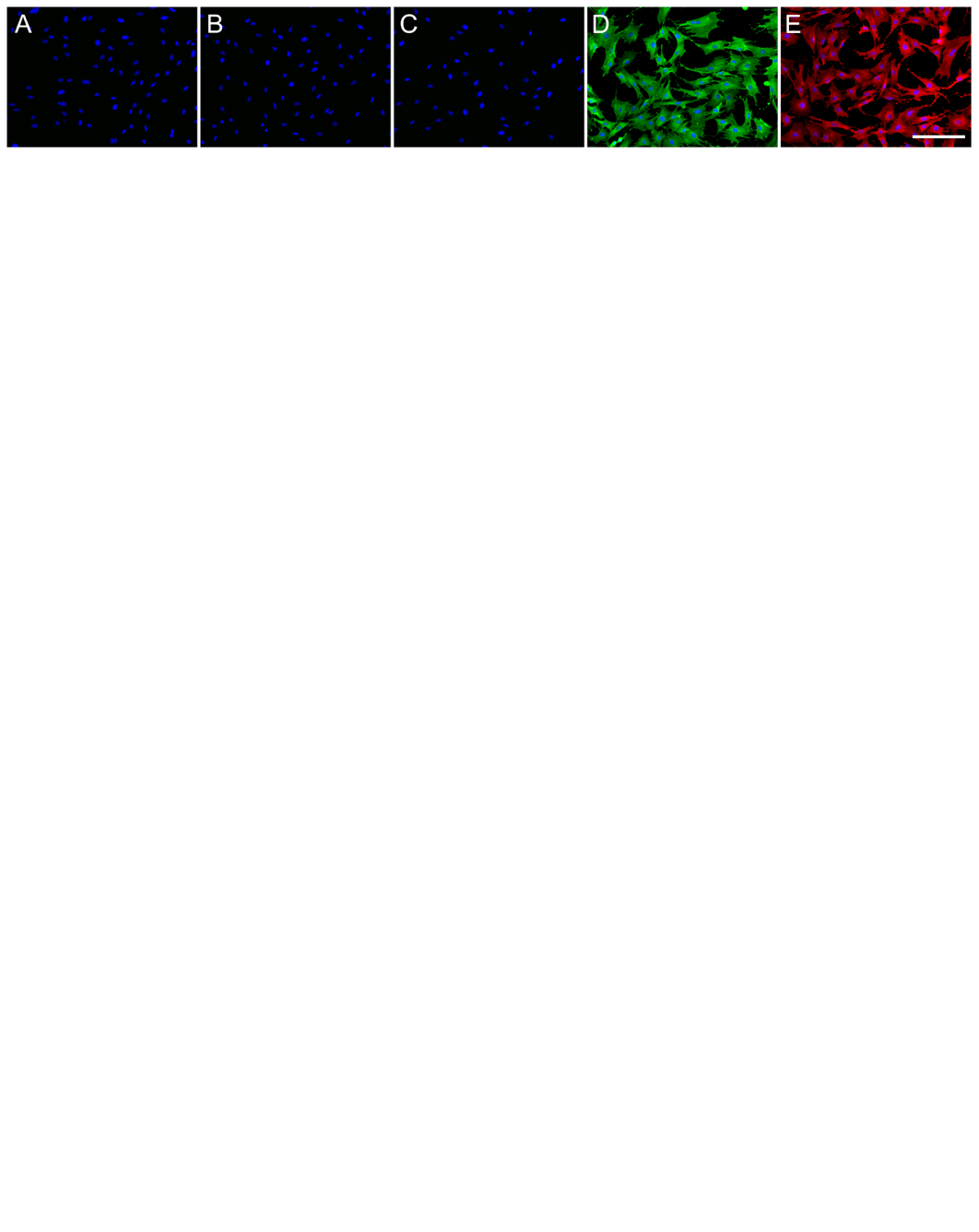
**

**Supplementary Fig.1 A** Negative control. **B** CD18 negative. **C** CD34 negative. **D** CD44 positive. **E** D90 positive. Scale Bars: 100μm.

**Supplementary Figure 2 ——Length of Native contralateral tendons of WT and ERβ^-/-^ mice**


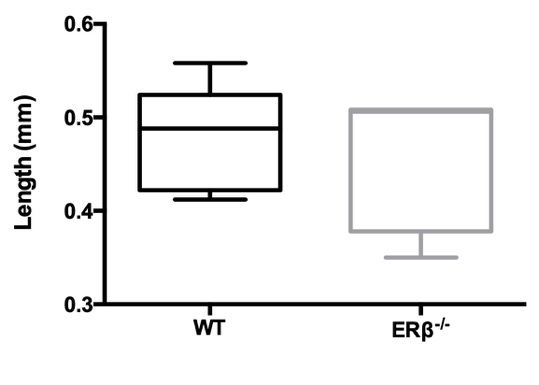


**Supplementary Fig.2** Length of native contralateral tendons of WT and ERβ^-/-^ mice. There is no significant difference of mean length of native contralateral tendons of WT and ERβ^-/-^ mice. The data are presented as the means ± s.d.s (n=5-8); p=0.6376.

**Supplementary Figure 3 —— Cross-sectional area of Native contralateral tendons of WT and ERβ^-/-^ mice**


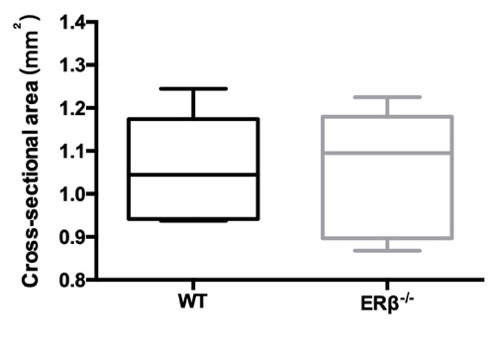


**Supplementary Fig.3** Cross-sectional area of native contralateral tendons of WT and ERβ^-/-^ mice. There is no significant difference of mean cross-sectional area of native contralateral tendons of WT and ERβ^-/-^ mice. The data are presented as the means ± s.d.s (n=5-8); p=0.8244.

**Supplementary Figure 4 —— Load to failure of Native contralateral tendons of WT and ERβ^-/-^ mice**


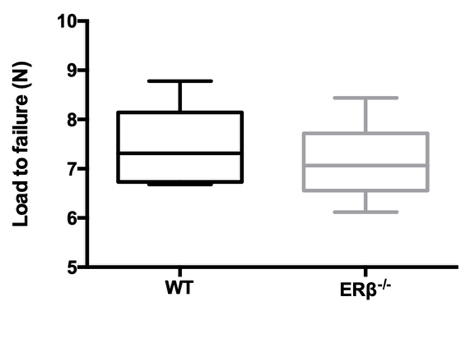


**Supplementary Fig.4** Failure to load of native contralateral tendons of WT and ERβ^-/-^ mice. There is no significant difference of mean failure to load of native contralateral tendons of WT and ERβ^-/-^ mice. The data are presented as the means ± s.d.s (n=5-8); p=0.5122.

**Supplementary Figure 5 —— Stiffness of Native contralateral tendons of WT and ERβ^-/-^ mice**


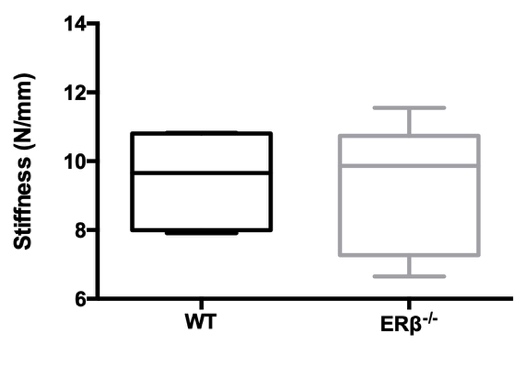


**Supplementary Fig.5** Stiffness of native contralateral tendons of WT and ERβ^-/-^ mice. There is no significant difference of mean stiffness of native contralateral tendons of WT and ERβ^-/-^ mice. The data are presented as the means ± s.d.s (n=5-8); p=0.8035.

**Supplementary Figure 6 —— Heatmap of correlation analysis of RNA sequencing between WT and ERβ^-/-^ mice**


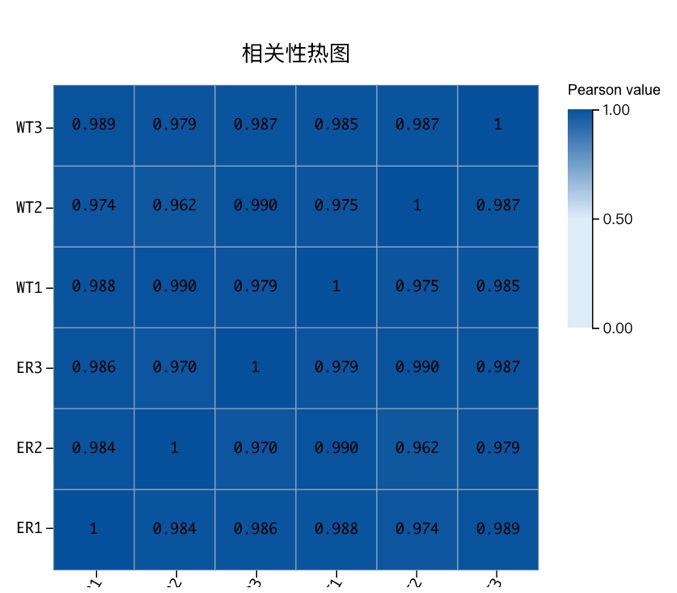


**Supplementary Fig.6** Stiffness of native contralateral tendons of WT and ERβ^-/-^ mice. There is no significant difference of mean stiffness of native contralateral tendons of WT and ERβ^-/-^ mice.

**Supplementary Table S1** Histological scoring system^1^

| Evaluated parameters | Points |
| --- | --- |
| *Extracellular matrix (ECM) organization of the whole tendon*  Wavy, compact and parallel arranged collagen fibers  In part compact, in part loose or not orderly  Loosely composed, not orderly (“granulation” tissue) | 2  1  0 |
| *Cellularity/cell-matrix-ratio*  Physiological  Locally increased cell density  Increased cell density or decreased ECM content | 2  1  0 |
| *Cell alignment*  Uniaxial  Areas of irregularly arranged cells (10-50%)  More than 50% of cells with no uniaxial alignment | 2  1  0 |
| *Cell distribution*  Homogeneous, physiological  Focal areas of elevated cell density (cell clustering) | 1  0 |
| *Cell nucleus morphology*  Predominantly elongated, heterochromatic cell nuclei (tenocytes)  10-30% of the cells possess large, oval, euchromatic or polymorph heterochromatic nuclei  Predominantly larger, oval, euchromatic or polymorph, heterochromatic nuclei | 2  1  0 |
| *Organization of repair tissue of the tendon callus*  Homogeneous (whole tissue with similar composition)  Locally heterogeneous tissue composition  Whole tissue composition completely changed | 2  1  0 |
| *Transition from defect to normal tissue*  Scaffold integrated, no gaps at the margin visible  Recognizable transition  Abrupt transition, splitting/gaps detectable, callus tissue | 2  1  0 |
| *Configuration of callus*  Normal, only in the defect area, locally confined  Strong, change of whole tendon, thickened | 1  0 |
| *Degenerative changes/tissue metaplasia*  Non existing  Moderate formation of oedema  Intense oedema with inclusion of fat, cell and/or fibers destruction, fibrin deposition, gaps  Assembly of cartilage or bone (Safranin Orange staining) | 3  2  1  0 |
| *Vascularization in the defect area*  Hypo-vascularized, like surrounding tendon (small capillaries)  Hyper-vascularized (increased numbers of small or larger capillaries) | 1  0 |
| *Inflammation*  No inflammatory cell infiltrates  Infiltrating inflammatory cell types (neutrophils, macrophages, foreign-body/giant cell) | 1  0 |

**Supplementary Table S2 qt-PCR Primers**

| Genes | Primer Sequences |
| --- | --- |
| Ms-FMOD | F:CACAACAGTCTCACTAACAACG  R:TCTTCTGCAGCTGATTGTAAGA |
| Ms-Col I | F:TGAACGTGGTGTACAAGGTC  R:CCATCTTTACCAGGAGAACCAT |
| Ms-Col Ⅲ | F:GAAAGAATGGGGAGACTGGAC  R:TACCAGGTATGCCTTGTAATCC |
| Ms-TNMD | F:GAACAGTCAGTGATTTGGGTTC  R:GGTCACATTATCGCAAATCTCC |
| GAPDH | F:CAAGGTCATCCATGACAACTTTG  R:GTCCACCACCCTGTTGCTGATG |

1. **Bian X, Liu T, Zhou M, He G, Ma Y, Shi Y, Wang Y, Tang H, Kang X, Yang M, Gustafsson JA, Fan X, Tang K.** Absence of estrogen receptor beta leads to abnormal adipogenesis during early tendon healing by an up-regulation of PPARgamma signalling. *J Cell Mol Med*. 2019.
